# Uncovering Potential lncRNAs and mRNAs in the Progression From Acute Myocardial Infarction to Myocardial Fibrosis to Heart Failure

**DOI:** 10.3389/fcvm.2021.664044

**Published:** 2021-07-16

**Authors:** Shuo Wang, Enmao Wang, Qincong Chen, Yan Yang, Lei Xu, Xiaolei Zhang, Rubing Wu, Xitian Hu, Zhihong Wu

**Affiliations:** Department of Cardiovasology, Shijiazhuang People's Hospital, Shijiazhuang, China

**Keywords:** acute myocardial infarction, myocardial fibrosis, heart failure, RNA sequencing, lncRNAs, mRNAs, signaling pathways, diagnosis

## Abstract

**Background:** Morbidity and mortality of heart failure (HF) post-myocardial infarction (MI) remain elevated. The aim of this study was to find potential long non-coding RNAs (lncRNAs) and mRNAs in the progression from acute myocardial infarction (AMI) to myocardial fibrosis (MF) to HF.

**Methods:** Firstly, blood samples from AMI, MF, and HF patients were used for RNA sequencing. Secondly, differentially expressed lncRNAs and mRNAs were obtained in MF vs. AMI and HF vs. MF, followed by functional analysis of shared differentially expressed mRNAs between two groups. Thirdly, interaction networks of lncRNA-nearby targeted mRNA and lncRNA-co-expressed mRNA were constructed in MF vs. AMI and HF vs. MF. Finally, expression validation and diagnostic capability analysis of selected lncRNAs and mRNAs were performed.

**Results:** Several lncRNA-co-expressed/nearby targeted mRNA pairs including AC005392.3/AC007278.2-IL18R1, AL356356.1/AL137145.2-PFKFB3, and MKNK1-AS1/LINC01127-IL1R2 were identified. Several signaling pathways including TNF and cytokine–cytokine receptor interaction, fructose and mannose metabolism and HIF-1, hematopoietic cell lineage and fluid shear stress, and atherosclerosis and estrogen were selected. IL1R2, IRAK3, LRG1, and PLAC4 had a potential diagnostic value for both AMI and HF.

**Conclusion:** Identified AC005392.3/AC007278.2-IL18R1, AL356356.1/AL137145.2-PFKFB3, and MKNK1-AS1/LINC01127-IL1R2 lncRNA-co-expressed/nearby targeted mRNA pairs may play crucial roles in the development of AMI, MF, and HF.

## Introduction

Heart failure (HF), the terminal stage of a wide range of cardiovascular diseases, leads to the decompensation of the heart's ability to contract or relax (1). In most clinical cases, HF is caused by myocardial infarction (MI). Following MI, a lot of cardiac cells will die in response to ischemia (2). As the adult heart has a very limited capacity to regenerate after injury, the lost cardiac cells will be replaced by fibrotic scars. The process is accompanied by remodeling of the surrounding myocardium and eventually results in impaired cardiac function. HF is significantly associated with cardiac hypertrophy, valvular disease, dilated cardiomyopathy, hypertrophic cardiomyopathy, age, obesity, diabetes, and hypertension (3, 4). Despite effective treatments targeted at improving HF, the 5-year mortality rate of the disease varies from 45 to 60% (5). Therefore, in-depth understanding of pathological mechanisms of HF is very important to develop effective therapy and assessment of patient prognosis.

Long non-coding RNAs (lncRNAs) regulate mRNA expression at the transcriptional and post-transcriptional levels. It has been shown that lncRNAs are emerging as essential players in heart development, atherosclerosis, and HF (6). In addition, the major component of HF disease management is diagnosis and monitoring of disease progression. In this study, we tried to find potential differentially expressed lncRNAs and mRNAs in the blood of patients with acute myocardial infarction (AMI), myocardial fibrosis (MF), and HF. We supposed that these potential differentially expressed lncRNAs and interacted mRNAs may be involved in the disease progression from AMI, MF and HF.

## Materials and Methods

### Subjects

In this study, three patients with AMI, three patients with MF, and three patients with HF were enrolled. The inclusion criteria of AMI patients were as follows: (1) time of chest pain or distress > 30 min within 24 h, and the level of the myocardial enzyme of creatine kinase (CK)-MB and cardiac troponin T (cTnT) was higher than the normal range; (2) patients had their first episode; (3) patients received no medical or surgical treatment prior to admission; (4) patients had blood samples before admission, at discharge, and 6 months after MI; and (5) patients had complete clinical data, including gender, age, height, and weight. The exclusion criteria of AMI patients were as follows: (1) patients with myocarditis and other diseases caused by chest pain or distress; (2) patients with a history of renal failure, advanced liver disease, malignant tumors, and other inflammatory diseases such as psoriasis and rheumatoid arthritis; (3) recurrent patients; (4) patients with incomplete clinical data; and (5) patients with missing blood samples before hospitalization, at discharge, and 6 months after MI. Those patients diagnosed with MF (1 year after MI) were enrolled. Depending on the diagnostic criteria, those patients with HF caused by MI were also enrolled.

### Blood Samples

The blood of AMI, MF, and HF patients was collected. Ethical approval was obtained from the ethics committee of the Shijiazhuang People's Hospital. In addition, informed written consent was obtained from all individuals.

### RNA Isolation and Sequencing

The total RNA was extracted from the blood sample using the RNAliquid Reagent according to the manufacturer's protocols. Agilent 2100 was utilized to test the RNA integrity number (RIN) (7). RNA conditions to be met for sequencing were as follows: (1) the total amount (required for single database construction) is 5 μg; (2) the concentration was ≥200 ng/μl; and (3) the value of OD260/280 was between 1.8 and 2.2. After total RNA DNase I digestion, removal of rRNA, RNA interruption, synthesis of reverse transcriptional one and two strands, end repair, cDNA with an “A” at the 3′ end, connection of cDNA 5′ adapter, digestion of two strands of cDNA, PCR reaction, and product recovery and library quality inspection, the Illumina Hiseq x-ten platform (PE150 strategy) (8) was used to perform RNA sequencing for lncRNA. After RNA fragment selection, 3' connector connection, reverse primer annealing, 5' connector connection, the synthesis of a strand of cDNA, PCR amplification, library fragment selection, library quantification, and pooling annularization, BGISEQ-500 platform (SE50 strategy) (9) was used to perform RNA sequencing for mRNA. The Combat function for the SVA package was used to eliminate batch effect (10). The Fastx-Toolkit was used to trim 5' and 3' segments of reads to remove bases with mass <20 and delete reads with N >10%. For lncRNA analysis, the cleaned gene sequencing reads were aligned to the reference genome (GRCh38) for matching *via* HISAT2 (11) (https://ccb.jhu.edu/software/hisat2/index.shtml). Stringtie (12) (http://www.ccb.jhu.edu/software/stringtie/) was utilized to quantify the expression levels of lncRNA and mRNA. For mRNA analysis, the Rfam (13) was used for annotation analysis on measured small RNA. The mature mRNA and mRNA precursor sequences were downloaded from miRBase (14). The expression of mRNA was quantified with miRDeep2 (15). Finally, DEGSeq2 package (16) was used to compare the expression difference of lncRNA and mRNA between the two groups in the R environment. The value of fragments per kilobase of exon per million mapped reads (FPKM) of each gene/transcript in the sample was calculated according to the comparison results of all samples with the reference genome. The value was regarded as the expression amount of gene/transcript in the sample.

### Identification of Differentially Expressed lncRNAs and mRNAs in MF vs. AMI and HF vs. MF

Differential expression analysis of lncRNAs and mRNAs in MF vs. AMI and HF vs. MF was evaluated in the R-bioconductor package DESeq2. Firstly, in order to normalize for sequencing depth, the original read count performed normalization. Then, the hypothesis testing probability (*p*-value) was obtained by the statistical model. Thirdly, multiple hypothesis test correction was carried out to obtain the corrected *p*-value [false discovery rate (FDR)]. Those differentially expressed lncRNAs and mRNAs were identified under the criterion of *p*-value < 0.05 and |log2Fold change| ≥ 1.

### Functional Analysis of Common Differentially Expressed mRNAs in MF vs. AMI and HF vs. MF

In order to study the biological function of common differentially expressed mRNAs in MF vs. AMI and HF vs. MF, functional analysis including Gene Ontology (GO) classification (17) and Kyoto Encyclopedia of Genes and Genomes (KEGG) pathway enrichment analysis (18) was performed through CPDB (http://cpdb.molgen.mpg.de/CPDB). Significantly enriched GO and KEGG terms were identified under the criterion of *p*-value < 0.05.

### Nearby Target and Co-expression Analysis Between lncRNA and mRNA in MF vs. AMI and HF vs. MF

In order to identify nearby targeted differentially expressed mRNAs of differentially expressed lncRNAs in MF vs. AMI and HF vs. MF, the mRNAs transcribed within a 100-kb window up- or downstream of lncRNAs (19) were searched by using BEDTools (20). In addition, the Pearson correlation coefficient (cor) of lncRNAs and mRNA was calculated using the cor.test function in R. The correlation pairs with |cor| ≥ 0.98 and *p*-value < 0.05 were considered in that lncRNAs were significantly co-expressed with mRNAs. Interaction networks of lncRNA-nearby mRNA and lncRNA-co-expressed mRNA were visualized by using Cytoscape (21).

### *In vitro* Validation of Key Differentially Expressed lncRNAs and mRNAs

In total, five patients with AMI (three patients from RNA sequencing), three patients with MF (two patients from RNA sequencing), and five patients with HF (three patients from RNA sequencing) were enrolled. The inclusion criteria of patients with AMI, MF, and HF were consistent with that for RNA sequencing. Ethical approval was obtained from the ethics committee of the Shijiazhuang People's Hospital and informed written consent was obtained from all individuals.

Total RNA of the blood samples was extracted according to the manufacturer's protocols. Then, qPCR was performed in an ABI 7300 qPCR system with SYBR® Green PCR Master Mix. All reactions were performed in triplicate. ACTB and GAPDH were used for the internal reference. Relative gene expression was analyzed by the fold change method. *p* < 0.05 is the significance level.

### Electronic Validation and Diagnostic Analysis of Key Differentially Expressed lncRNAs and mRNAs

In order to further validate the expression of the identified key differentially expressed lncRNAs and mRNAs, the GSE21125 dataset (HF cases:normal controls = 18:9) and the GSE48060 dataset (AMI cases:normal controls = 31:21) were used for expression validation. The expression pattern was displayed in the box plots. In addition, we also performed the ROC analyses to assess the diagnostic value of lncRNAs and mRNAs in the above two datasets.

## Results

### Clinical Information of Enrolled Individuals for RNA Sequencing and *in vitro* Validation

A total of 14 patients (5 AMI patients, 4 MF patients, and 5 HF patients) were enrolled in both RNA sequencing and *in vitro* validation. The clinical information of these individuals is presented in Table 1. The chisq.test of gender showed that there was no statistical difference of gender between male and female patients in three groups (*p*-value = 0.638).

**Table 1:** The clinical information of all individuals enrolled in RNA sequencing and *in vitro* validation.

**Group**	**Gender**	**Age**	**Height (cm)**	**Weight (kg)**	**BMI**	**Hypertension history**	**Diabetes history**	**Smoking history**	**Drinking history**	**Cerebrovascular disease history**	**Family history of CHD**	**Hemoglobin (g/L)**	**LDL (mmol/L)**	**HDL (mmol/L)**	**TC (mmol/L)**	**TG (mmol/L)**	**Auscultation situation**	**Electrocardiogram**	**Cardiac uhrasonography**
AMI	Female	81	158	56	23	Yes	No	No	No	No	No	127	3.04	0.82	4.25	1.61	Not noise	Third degree atrioventricular block, ST segment elevation of lower wall lead 0.3mV.	Segmental wall motion abnormality (left anterior wall movement is not coordinated, lower wall movement is weakened), LVEF58%.
	Male	54	175	80	26	Yes	No	No	No	No	No	151	2.75	1.21	4.2	1.08	Not noise	Sinus rhythm, lead V1-5 QS type, ST-segment elevation 0.4mV.	Segmental wall motion abnormality
	Female	80	160	58	23	No	Yes	No	No	No	No	119	2.53	1.18	3.98	0.88	Slight wet rales can be heard at the base of both lungs	ST segment elevation in lead V1-V6	Left ventricular systolic function decreased
	Male	56	173	75	25	Yes	Yes	No	No	No	No	152	3.51	0.82	5.05	3.07	Not dry or wet rales	The anterior E peak of the intervalve blood flow decreased	The mitral valve can detect trace regurgitation signal
	Male	65	170	80	27.68	Yes	Yes	No	No	No	No	143	3.49	0.89	5.04	1.88	Not dry or wet rales	arrhythmia	Bilateral enlargement showed abnormal segmental ventricular wall motion, slight insufficiency of the second and third valves, and reduced left ventricular function.
MF	Male	71	170	80	27.68	Yes	No	No	No	No	No	147	4.59	1.47	6.91	0.94	Not dry or wet rales	Segmental wall movement is abnormal	Left ventricular dysfunction
	Male	66	175	78	25.47	Yes	No	Yes	No	Yes	No	156	2.43	0.94	3.78	2.21	Slight wet rales can be heard at the base of both lungs	The front wall lead T wave is inverted	The left ventricular wall motion amplitude of LA40 and LV66 was generally reduced, with the exception of the formation of ventricular aneurysm from the anterior wall to the apex of the heart, mild aortic valve insufficiency, minor mitral and tricuspid valve insufficiency, and LVEF43%.
	Male	51	173	59	19.71	No	No	Yes	Yes	No	No	151	2.56	1.14	3.92	1.15	Not dry or wet rales	Sinus rhythm	Tricuspid valve mild insufficiency, Lvef 47%.
	Female	54	164	58	21.56	No	Yes	No	No	No	No	138	2.34	1.08	3.79	0.64	Not dry or wet rales	Sinus rhythm, anterior wall lead T wave is low and flat.	Segmental wall motion abnormality
HF	Female	79	160	80	31.25	Yes	Yes	No	No	No	No	99	2.58	0.78	3.74	1.94	Wet rales all over the lungs	ST segment elevation in the front wall lead	LA43mm, LV57mm, segmental ventricular wall motion abnormality, LVEF31%.
	Male	73	180	75	23.15	Yes	Yes	Yes	Yes	No	No	122	1.62	0.79	2.63	0.67	Not rales	Sinus rhythm, anterior wall lead T - wave inversion.	Segmental wall motion abnormality
	Male	82	170	66	22.84	Yes	No	No	No	No	No	147	2.2	1.12	3.52	1.11	Wet rales can be heard in both lungs	Pace rhythm, third degree atrioventricular block.	Enlargement of left heart, segmental ventricular wall motility disorder, pulmonary hypertension (mild), Lvef42%.
	Female	69	160	65	25.39	Yes	No	No	No	No	No	113.8	2.97	0.45	5.95	3.04	Not dry or wet rales	Increased left heart	Cardiac dysfunction
	Female	80	163	68	25.59	Yes	Yes	No	No	No	No	126	1.57	1.01	2.92	1.09	Wet rales	Sinus rhythm, V2-V6 lead ST segment depression, AVR elevation.	LV52mm, abnormal segmental ventricular wall motion, LVEF38%.

*AMI, acute myocardial infarction; MF, myocardial fibrosis; HF, heart failure; CHD, coronary heart disease; BMI, body mass index; LDL, low density lipoprotein; HDL, high density lipoprotein; TC, total cholesterol; TG, triglyceride*.

*Samples with red font is used for RNA-seq; samples with highlighted in yellow is used for qPCR*.

### Differentially Expressed lncRNAs and mRNAs in MF vs. AMI and HF vs. MF

**Figure 1:**
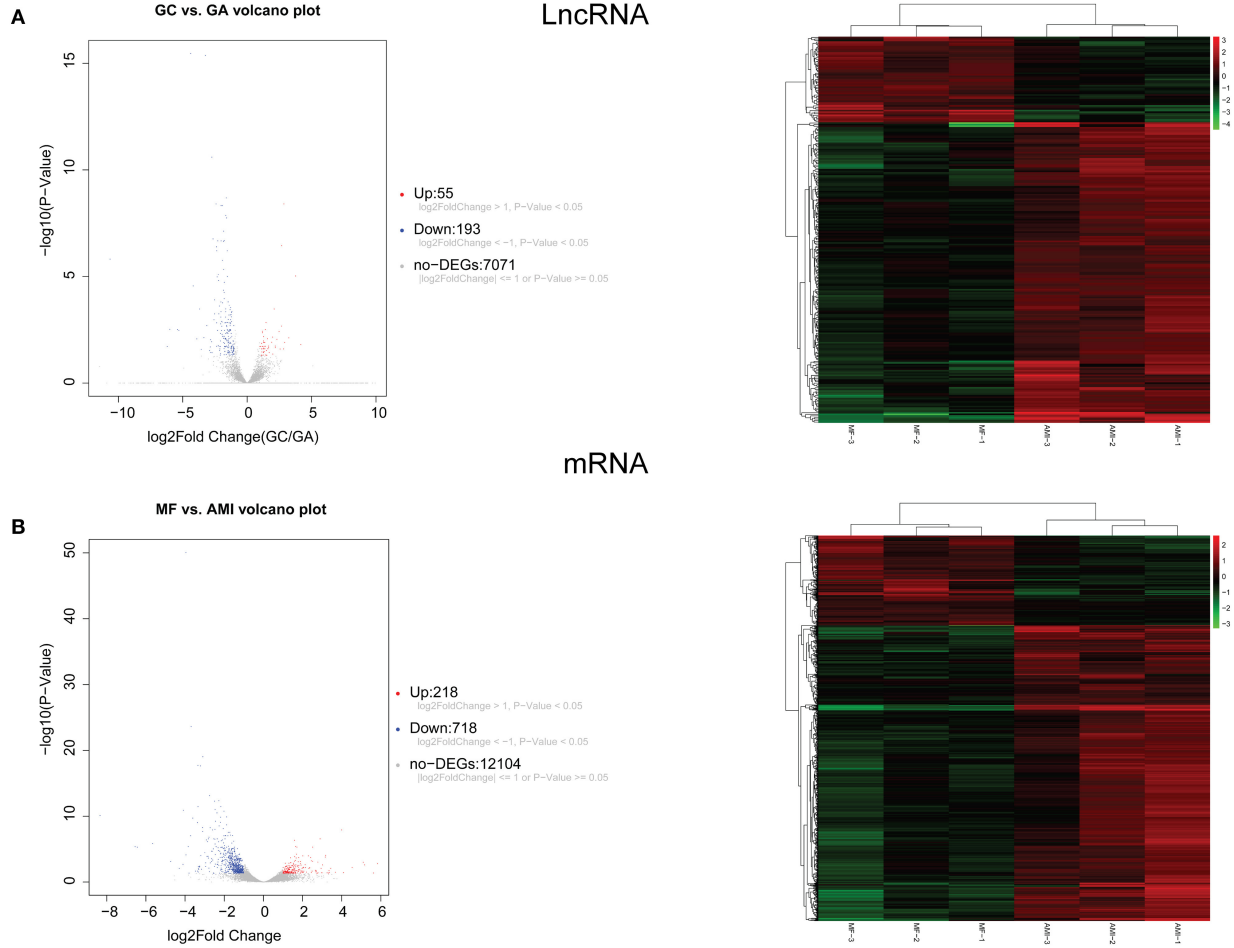
The volcano plot and hierarchical clustering diagram of lncRNAs **(A)** and mRNAs **(B)** in MF vs. AMI. In the volcano, the *x*-axis and *y*-axis present Log2 fold change and –log10 *p*-value, respectively. Red and blue color represent upregulation and downregulation, respectively. In the hierarchical clustering diagram, the diagram presents the result of a two-way hierarchical clustering of lncRNAs/mRNAs and sample. Each row represents a differentially expressed lncRNA/mRNA, and each column represents a sample. Differentially expressed lncRNAs/mRNAs clustering tree is shown on the top right corner. The color scale illustrates the relative level of differentially expressed genes: red, below the reference channel; green, higher than the reference.

**Figure 2:**
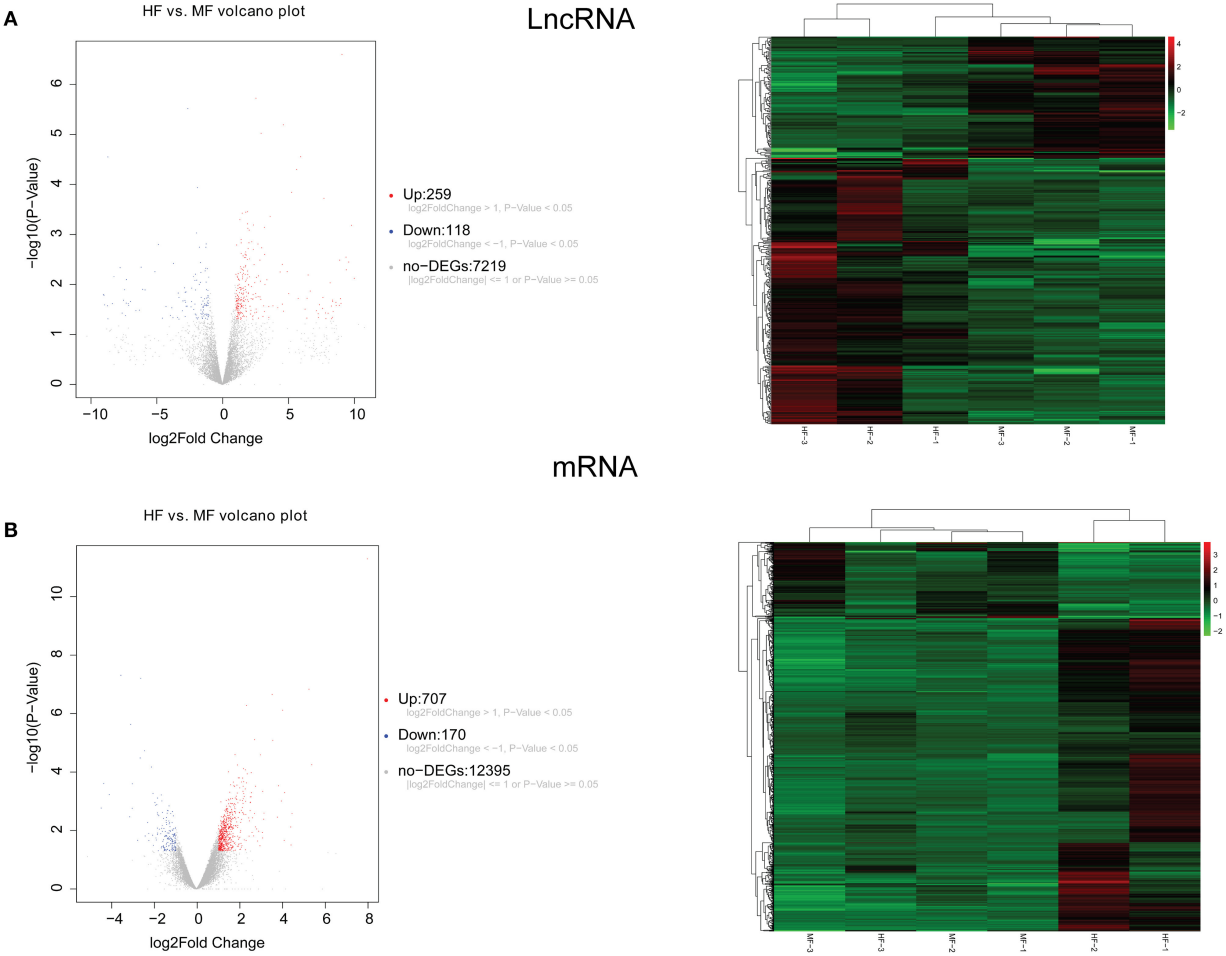
The volcano plot and hierarchical clustering diagram of lncRNAs **(A)** and mRNAs **(B)** in HF vs. MF. In the volcano, the *x*-axis and *y*-axis present Log2 fold change and –log10 *p*-value, respectively. Red and blue color represent upregulation and downregulation, respectively. In the hierarchical clustering diagram, the diagram presents the result of a two-way hierarchical clustering of lncRNAs/mRNAs and sample. Each row represents a differentially expressed lncRNA/mRNA, and each column represents a sample. Differentially expressed lncRNAs/mRNAs clustering tree is shown on the top right corner. The color scale illustrates the relative level of differentially expressed genes: red, below the reference channel; green, higher than the reference.

A total of 248 differentially expressed lncRNAs (Supplementary Table 1) and 936 differentially expressed mRNAs (Supplementary Table 2) were obtained in MF vs. AMI. The volcano plot and hierarchical clustering diagram of lncRNAs and mRNAs are shown in Figure 1. The top 10 up- and downregulated differentially expressed lncRNAs and mRNAs are shown in Tables 2, 3, respectively. In addition, a total of 377 differentially expressed lncRNAs (Supplementary Table 3) and 877 differentially expressed mRNAs (Supplementary Table 4) were obtained in HF vs. MF. The volcano plot and hierarchical clustering diagram of lncRNAs and mRNAs are shown in Figure 2. In addition, the top 10 up- and downregulated differentially expressed lncRNAs and mRNAs are listed in Tables 4, 5, respectively. Interestingly, a total of 87 common differentially expressed lncRNAs (such as AC005392.3, AC007278.2, AL356356.1, AL137145.2, MKNK1-AS1, LINC01127, and PLAC4) and 341 differentially expressed mRNAs (such as IL18R1, PFKFB3, IL1R2, IRAK3, FKBP5, LRG1, IKZF2, and RNASE1) were identified between MF vs. AMI and HF vs. MF. Beside PLAC4 and IKZF2, all above common lncRNAs and mRNAs were downregulated in MF vs. AMI, while they were upregulated in HF vs. MF. On the contrary, PLAC4 and IKZF2 were upregulated in MF vs. AMI, while they were downregulated in HF vs. MF.

**Table 2:** Top 10 up- and down-regulated differentially expressed lncRNAs in MF vs. AMI.

**LncRNA ID**	**LncRNA name**	**Log2FC**	**P value**	**Regulation**
ENSG00000244879	GABPB1-AS1	2.863568	5.63E-12	Up
ENSG00000253522	MIR3142HG	2.689237	1.45E-09	Up
ENSG00000250696	AC111000.4	3.770192	5.64E-08	Up
ENSG00000280109	PLAC4	2.108987	3.44E-06	Up
ENSG00000267349	AC015911.5	1.474202	2.22E-05	Up
ENSG00000254363	AC011379.2	2.677708	3.90E-05	Up
ENSG00000267074	AC015911.3	1.423765	6.10E-05	Up
ENSG00000248049	UBA6-AS1	1.194013	8.38E-05	Up
ENSG00000284734	AC099063.4	2.486121	8.94E-05	Up
ENSG00000225434	LINC01504	1.203724	0.000103	Up
ENSG00000258831	AC103996.2	−4.36855	8.33E-20	Down
ENSG00000258476	LINC02207	−3.22901	2.05E-19	Down
ENSG00000203999	LINC01270	−2.72995	1.81E-14	Down
ENSG00000179406	LINC00174	−1.60748	1.96E-12	Down
ENSG00000281162	LINC01127	−2.40124	5.26E-12	Down
ENSG00000256072	AC078889.1	−2.01354	9.04E-12	Down
ENSG00000212743	AL137145.1	−1.92348	9.10E-12	Down
ENSG00000269956	MKNK1-AS1	−1.64687	2.96E-11	Down
ENSG00000284930	AC005280.2	−1.58936	4.25E-11	Down
ENSG00000279511	AL356274.2	−1.81148	1.96E-10	Down

*FC, fold change*.

**Table 3:** Top 10 up- and down-regulated differentially expressed mRNAs in MF vs. AMI.

**mRNA ID**	**mRNA name**	**Log2FC**	***P*-value**	**Regulation**
ENSG00000144290	SLC4A10	4.004457	2.71E-11	Up
ENSG00000103056	SMPD3	2.901452	9.39E-10	Up
ENSG00000116747	TROVE2	1.599512	1.71E-09	Up
ENSG00000160613	PCSK7	1.652641	1.94E-08	Up
ENSG00000163606	CD200R1	1.719633	2.46E-08	Up
ENSG00000167635	ZNF146	2.523442	2.50E-08	Up
ENSG00000198780	FAM169A	1.698022	5.60E-07	Up
ENSG00000134489	HRH4	3.188331	8.94E-07	Up
ENSG00000113088	GZMK	2.304332	1.14E-06	Up
ENSG00000030419	IKZF2	2.017825	1.15E-06	Up
ENSG00000115604	IL18R1	−3.94927	6.87E-55	Down
ENSG00000169902	TPST1	−3.68416	3.80E-28	Down
ENSG00000170525	PFKFB3	−3.08449	2.12E-23	Down
ENSG00000123836	PFKFB2	−3.33753	5.92E-22	Down
ENSG00000096060	FKBP5	−3.20944	8.85E-22	Down
ENSG00000115590	IL1R2	−2.74328	3.53E-17	Down
ENSG00000171236	LRG1	−2.26774	2.38E-16	Down
ENSG00000183019	MCEMP1	−2.48928	3.20E-16	Down
ENSG00000090376	IRAK3	−2.16585	2.47E-15	Down
ENSG00000106714	CNTNAP3	−3.33164	2.65E-15	Down

*FC, fold change*.

**Table 4:** Top 10 up- and down-regulated differentially expressed lncRNAs in HF vs. MF.

**LncRNA ID**	**LncRNA name**	**Log2FC**	***P*-value**	**Regulation**
ENSG00000280441	FP236383.1	9.063356	2.51E-07	Up
ENSG00000203999	LINC01270	2.533682	1.88E-06	Up
ENSG00000234665	AL512625.3	4.609455	6.42E-06	Up
ENSG00000261519	AC010542.4	2.924686	9.51E-06	Up
ENSG00000251129	LINC02506	5.932828	2.80E-05	Up
ENSG00000259309	AC021818.1	5.62467	5.02E-05	Up
ENSG00000260176	AC141586.2	5.241057	0.000144	Up
ENSG00000285957	AC019257.7	7.696822	0.000189	Up
ENSG00000261971	MMP25-AS1	1.887239	0.000352	Up
ENSG00000237781	AL356356.1	1.765794	0.000358	Up
ENSG00000244879	GABPB1-AS1	−2.63555	3.05E-06	Down
ENSG00000241280	AC106712.1	−8.7057	2.83E-05	Down
ENSG00000280109	PLAC4	−1.90798	0.000116	Down
ENSG00000250696	AC111000.4	−1.9815	0.000935	Down
ENSG00000279364	AC114546.3	−1.24751	0.001545	Down
ENSG00000260412	AL353746.1	−4.87421	0.001587	Down
ENSG00000273448	AC006480.2	−1.7627	0.001797	Down
ENSG00000259623	AC125257.1	−1.26554	0.001804	Down
ENSG00000255733	IFNG-AS1	−1.6751	0.003644	Down
ENSG00000266680	AL135905.1	−3.72605	0.003799	Down

*FC, fold change*.

**Table 5:** Top 10 up- and down-regulated differentially expressed mRNAs in HF vs. MF.

**mRNA ID**	**mRNA name**	**Log2FC**	**P value**	**Regulation**
ENSG00000285953	AC000120.3	7.970556	5.11E-12	Up
ENSG00000087116	ADAMTS2	5.240814	1.47E-07	Up
ENSG00000168916	ZNF608	3.514254	2.20E-07	Up
ENSG00000206053	JPT2	2.311437	5.30E-07	Up
ENSG00000239704	CDRT4	4.012593	7.66E-07	Up
ENSG00000188897	AC099489.1	2.692945	7.84E-06	Up
ENSG00000246922	UBAP1L	3.546788	8.22E-06	Up
ENSG00000007545	CRAMP1	1.778259	2.54E-05	Up
ENSG00000169902	TPST1	2.964558	2.58E-05	Up
ENSG00000129538	RNASE1	5.371539	5.62E-05	Up
ENSG00000259024	TVP23C-CDRT4	−3.56763	4.89E-08	Down
ENSG00000139351	SYCP3	−2.63416	6.25E-08	Down
ENSG00000258790	AL121594.1	−3.10555	2.35E-06	Down
ENSG00000198574	SH2D1B	−2.46177	1.87E-05	Down
ENSG00000131203	IDO1	−2.66387	3.29E-05	Down
ENSG00000134545	KLRC1	−2.13404	6.71E-05	Down
ENSG00000160307	S100B	−4.37072	0.000248	Down
ENSG00000214756	CSKMT	−3.03242	0.000253	Down
ENSG00000234284	ZNF879	−2.06443	0.000541	Down
ENSG00000203989	RHOXF2B	−4.11115	0.000588	Down

*FC, fold change*.

### Functional Analysis of Common Differentially Expressed mRNAs Between MF vs. AMI and HF vs. MF

**Figure 3:**
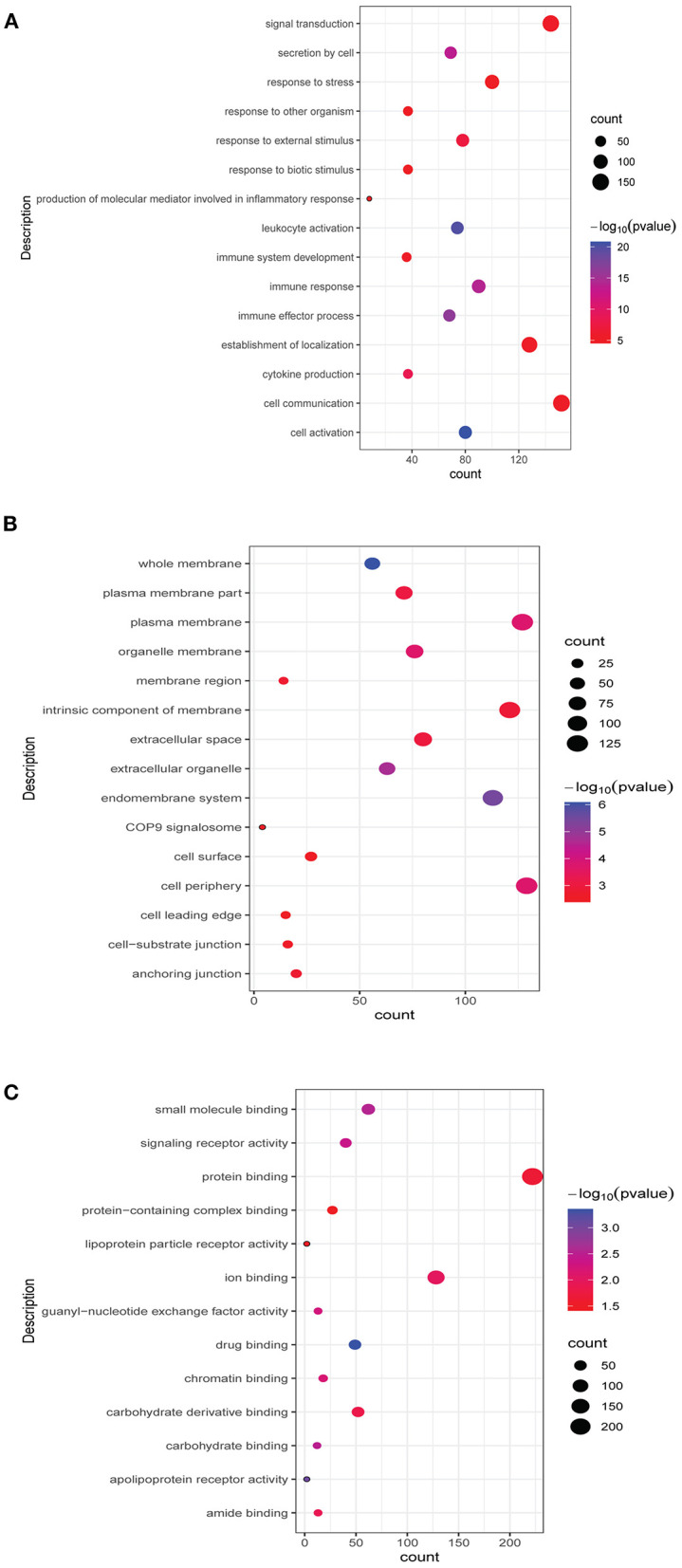
Top 15 significant enrichment GO terms of common differentially expressed mRNAs between MF vs. AMI and HF vs. MF. The *x*-axis and *y*-axis present the number of enriching mRNAs and GO terms, respectively. **(A)**, biological progress; **(B)**, cellular component; **(C)**, molecular function.

Functional analysis of common differentially expressed mRNAs between MF vs. AMI and HF vs. MF showed that cell activation, whole membrane, and drug binding were the most significantly enriched GO terms (Figure 3). In addition, several enriched signaling pathways in the KEGG analysis were identified, including TNF signaling pathway and cytokine–cytokine receptor interaction (involving IL18R1), fructose and mannose metabolism and HIF-1 signaling pathway (involving PFKFB3), hematopoietic cell lineage and fluid shear stress and atherosclerosis (involving IL1R2), and estrogen signaling pathway (involving FKBP5). All significantly enriched KEGG signaling pathways and involved mRNAs are listed in Table 6.

**Table 6:** All significantly enriched KEGG signaling pathways of common differentially expressed mRNAs between MF vs. AMI and HF vs. MF.

**ID**	**Description**	**mRNA ID**	**Count**	***P*-value**
hsa04640	Hematopoietic cell lineage	HLA-DPB1/IL5RA/CR1/IL4R/IL1R2/ITGAM/CSF3R/HLA-DPA1/FLT3/IL1R1	10	2.08E-05
hsa05202	Transcriptional misregulation in cancer	RARA/CEBPB/IGF1R/SPI1/CXCL8/MLLT1/FLT3/DEFA3/ITGAM/RXRA/MMP9/BCL6/IL1R2	13	8.58E-05
hsa05140	Leishmaniasis	HLA-DPB1/NCF1/CR1/NCF4/TLR2/ITGAM/MAPK13/HLA-DPA1	8	9.82E-05
hsa05152	Tuberculosis	CEBPB/CR1/HLA-DPB1/PLK3/MAPK13/CTSD/TCIRG1/ITGAM/NOD2/HLA-DPA1/TLR2/LSP1	12	0.000236
hsa04659	Th17 cell differentiation	RARA/HLA-DPB1/IL4R/RXRA/STAT5A/JAK3/MAPK13/HLA-DPA1/IL1R1	9	0.000283
hsa04658	Th1 and Th2 cell differentiation	HLA-DPB1/MAML3/NOTCH1/STAT5A/JAK3/MAPK13/HLA-DPA1/IL4R	8	0.000491
hsa05134	Legionellosis	CR1/HSPA6/CXCL8/TLR2/HSPA1B/ITGAM	6	0.000757
hsa04380	Osteoclast differentiation	NCF1/SPI1/SOCS3/SIRPA/NCF4/JUNB/MAPK13/LILRA5/IL1R1	9	0.000991
hsa04668	TNF signaling pathway	CEBPB/SOCS3/JUNB/IL18R1/MAPK13/CFLAR/NOD2/MMP9	8	0.001591
hsa04670	Leukocyte transendothelial migration	NCF1/NCF4/MAPK13/RASSF5/ACTN1/PXN/MMP9/ITGAM	8	0.001785
hsa05321	Inflammatory bowel disease (IBD)	HLA-DPB1/IL4R/IL18R1/TLR2/NOD2/HLA-DPA1	6	0.001829
hsa04930	Type II diabetes mellitus	HK3/PRKCZ/IRS2/CACNA1E/SOCS3	5	0.00213
hsa05144	Malaria	KLRK1/TLR2/CR1/KLRB1/CXCL8	5	0.002823
hsa00052	Galactose metabolism	HK3/MGAM/MGAM2/GAA	4	0.003178
hsa05200	Pathways in cancer	ADCY4/CSF2RB/IGF1R/SPI1/CSF3R/ARHGEF11/IL5RA/CXCL8/RASSF5/NOTCH1/RARA/GNB2/STAT5A/GLI1/RALB/JAK3/RXRA/MMP9/FLT3/IL4R	20	0.004359
hsa05166	Human T-cell leukemia virus 1 infection	ADCY4/HLA-DPB1/SPI1/ZFP36/TLN1/ETS2/STAT5A/JAK3/HLA-DPA1/IL1R2/IL1R1	11	0.00451
hsa04062	Chemokine signaling pathway	ADCY4/NCF1/PREX1/GRK6/CXCL8/GNB2/PRKCZ/JAK3/WAS/PXN	10	0.004638
hsa05150	Staphylococcus aureus infection	FCAR/HLA-DPA1/FPR2/ITGAM/HLA-DPB1	5	0.004676
hsa00500	Starch and sucrose metabolism	HK3/MGAM/MGAM2/GAA	4	0.005505
hsa05145	Toxoplasmosis	HLA-DPB1/HSPA6/ALOX5/TLR2/HSPA1B/MAPK13/HLA-DPA1	7	0.007426
hsa04213	Longevity regulating pathway - multiple species	ADCY4/HSPA1B/IGF1R/HSPA6/IRS2	5	0.007775
hsa05131	Shigellosis	CXCL8/NOD2/WAS/ARPC5/MAPK13	5	0.009459
hsa04144	Endocytosis	SMAP2/HSPA6/ARAP1/GRK6/IGF1R/ARPC5/HSPA1B/PRKCZ/IQSEC1/WAS/FOLR3	11	0.009876
hsa05221	Acute myeloid leukemia	RARA/STAT5A/SPI1/FLT3/ITGAM	5	0.010073
hsa04145	Phagosome	FCAR/HLA-DPB1/NCF1/NCF4/TCIRG1/TLR2/ITGAM/HLA-DPA1	8	0.010779
hsa05146	Amoebiasis	ACTN1/CXCL8/TLR2/ITGAM/IL1R2/IL1R1	6	0.011757
hsa04140	Autophagy - animal	IGF1R/ULK1/WIPI2/RRAS2/CTSD/IRS2/CFLAR	7	0.014168
hsa04630	JAK-STAT signaling pathway	CSF2RB/IL5RA/IL4R/SOCS3/STAT5A/OSM/JAK3/CSF3R	8	0.015934
hsa04913	Ovarian steroidogenesis	ADCY4/IGF1R/ALOX5/HSD17B2	4	0.016201
hsa04915	Estrogen signaling pathway	ADCY4/RARA/HSPA6/HSPA1B/CTSD/FKBP5/MMP9	7	0.019162
hsa05418	Fluid shear stress and atherosclerosis	NCF1/MAPK13/PRKCZ/MMP9/THBD/IL1R2/IL1R1	7	0.021319
hsa05164	Influenza A	HLA-DPB1/HSPA6/SOCS3/CXCL8/HSPA1B/MAPK13/HLA-DPA1/FURIN	8	0.02132
hsa04015	Rap1 signaling pathway	ADCY4/IGF1R/RASSF5/TLN1/PRKCZ/ITGAM/MAPK13/SIPA1L2/RALB	9	0.022435
hsa04360	Axon guidance	EPHB4/NTNG2/LRRC4/PLXNA2/UNC5A/PRKCZ/FES/SEMA4B	8	0.024087
hsa04810	Regulation of actin cytoskeleton	ACTN1/RRAS2/FGD3/ITGB8/MYH9/ARPC5/ITGAM/WAS/PXN	9	0.02709
hsa00051	Fructose and mannose metabolism	HK3/PFKFB3/PFKFB4	3	0.027507
hsa05132	Salmonella infection	CXCL8/MYH9/WAS/ARPC5/MAPK13	5	0.028571
hsa05323	Rheumatoid arthritis	HLA-DPA1/HLA-DPB1/TLR2/TCIRG1/CXCL8	5	0.032478
hsa04060	Cytokine-cytokine receptor interaction	CSF2RB/IL5RA/IL4R/CXCL8/IL18R1/TNFRSF10C/OSM/IL17RA/CSF3R/IL1R2/IL1R1	11	0.033849
hsa04657	IL-17 signaling pathway	CEBPB/CXCL8/MAPK13/IL17RA/MMP9	5	0.038184
hsa05223	Non-small cell lung cancer	STAT5A/RASSF5/JAK3/RXRA	4	0.042512
hsa04933	AGE-RAGE signaling pathway in diabetic complications	CXCL8/STAT5A/PRKCZ/THBD/MAPK13	5	0.047826
hsa05322	Systemic lupus erythematosus	HLA-DPB1/ACTN1/HIST1H2BE/HLA-DPA1/HIST1H2AD/TROVE2	6	0.048665
hsa04920	Adipocytokine signaling pathway	ACSL1/IRS2/RXRA/SOCS3	4	0.048728
hsa04066	HIF-1 signaling pathway	HK3/PFKFB3/MKNK1/IGF1R/TIMP1	5	0.049561

### Nearby Target and Co-expressed Analysis Between lncRNAs and mRNAs in MF vs. AMI and HF vs. MF

**Figure 4:**
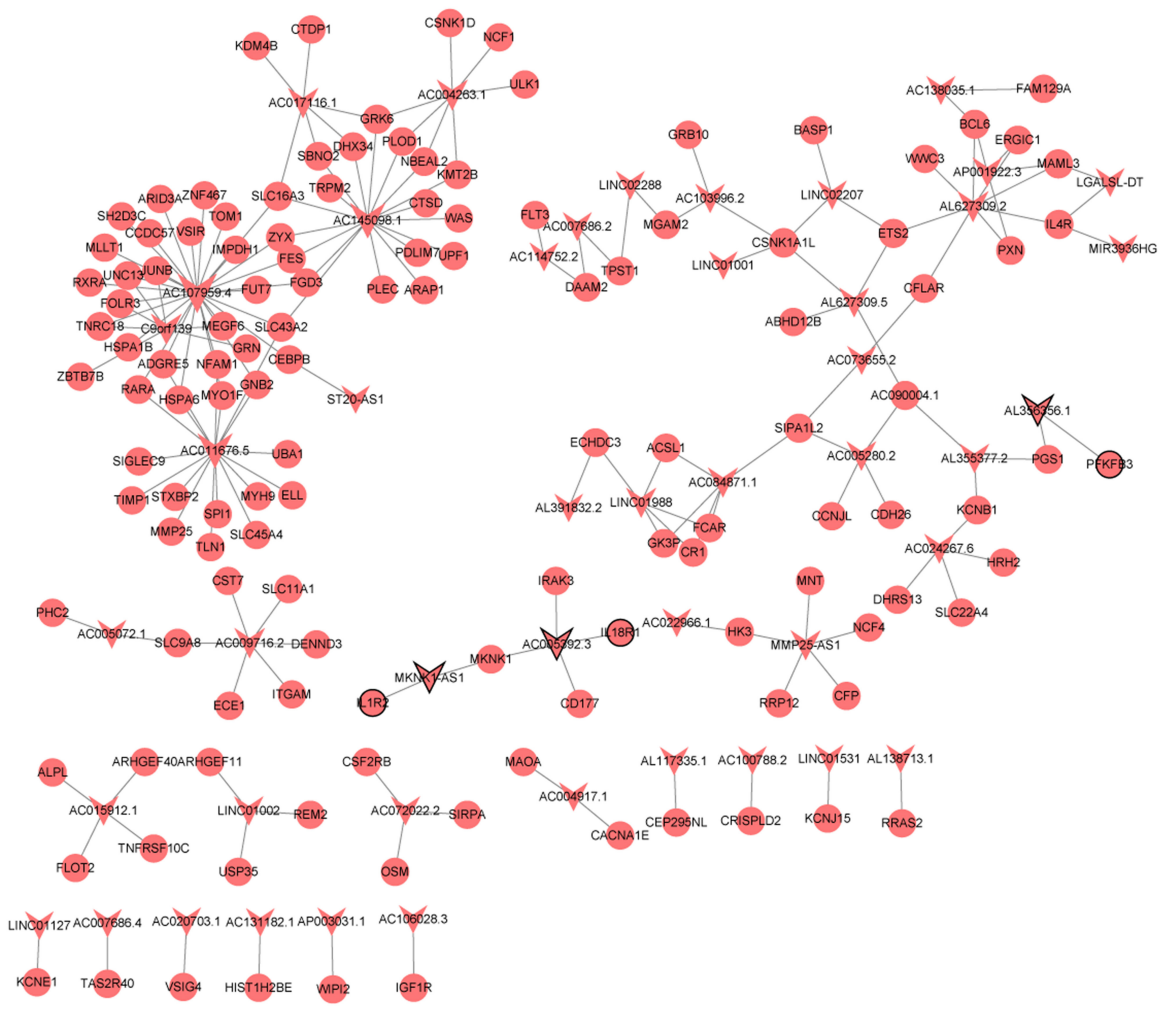
The interaction network of common lncRNA-co-expressed mRNA pairs between MF vs. AMI and HF vs. MF. The arrow and circle represent lncRNA and co-expressed mRNA, respectively. Arrow/circle with a black border represents core lncRNA/mRNA.

A total of 209 lncRNA-nearby targeted mRNA pairs (including 150 lncRNA and 155 mRNAs) and 171 lncRNA-nearby targeted mRNA pairs (including 109 lncRNA and 139 mRNAs) were obtained in MF vs. AMI and HF vs. MF, respectively. Interestingly, some common lncRNA-nearby targeted mRNA pairs between MF vs. AMI and HF vs. MF were found, such as AC007278.2-IL18R1, AL137145.2-PFKFB3, and LINC01127-IL1R2. In addition, a total of 1214 lncRNA-co-expressed mRNA pairs (including 80 lncRNA and 300 mRNAs) and 2069 lncRNA-co-expressed mRNA pairs (including 78 lncRNA and 288 mRNAs) were obtained in MF vs. AMI and HF vs. MF, respectively. Significantly, a total of 186 common lncRNA-co-expressed mRNA pairs were identified between MF vs. AMI and HF vs. MF. For instance, AC005392.3-IL18R1, AL356356.1-PFKFB3, and MKNK1-AS1-IL1R2 were found. The interaction network of common lncRNA-co-expressed mRNA pairs between MF vs. AMI and HF vs. MF is shown in Figure 4.

### *In vitro* Validation

**Figure 5:**
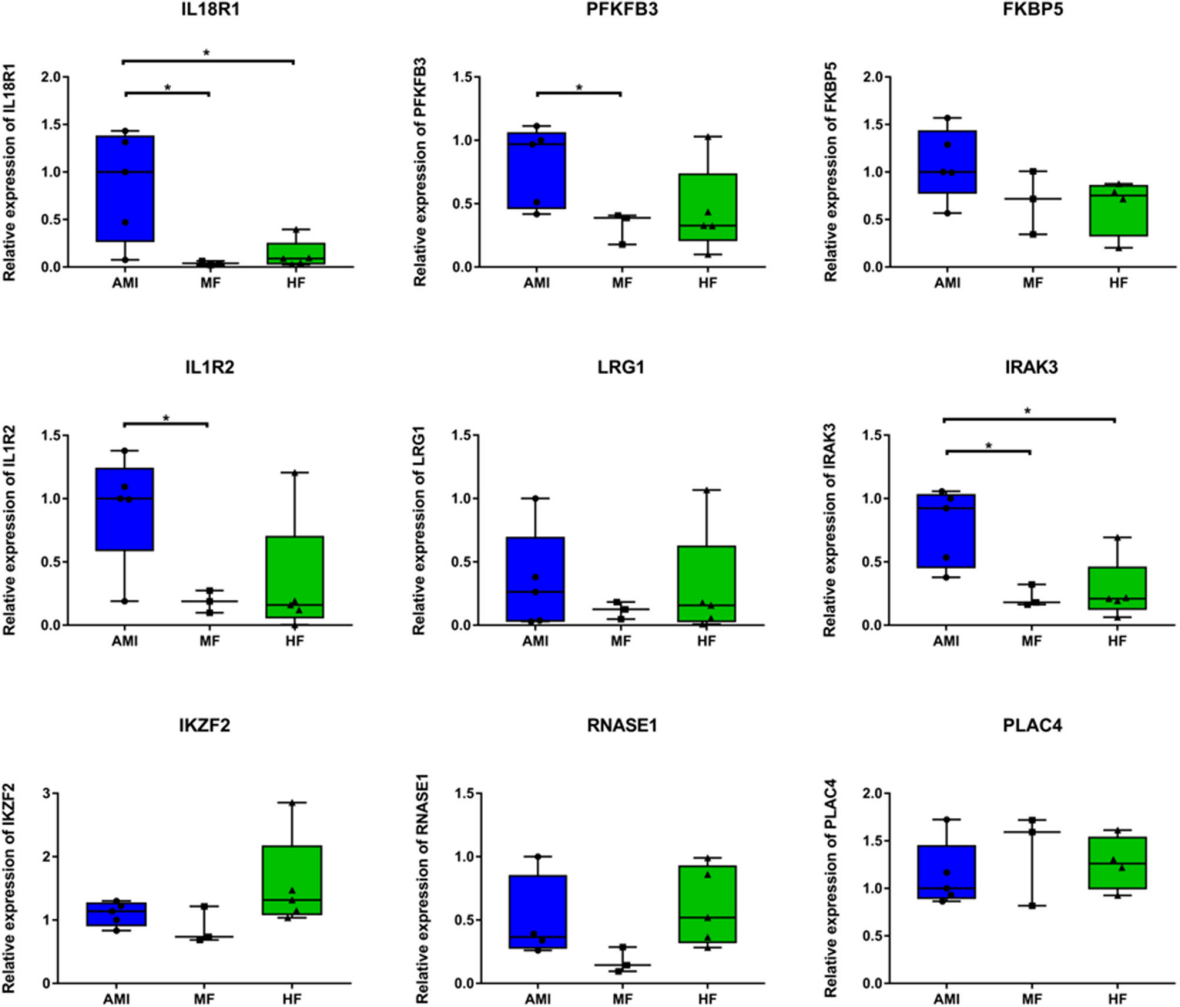
The *in vitro* qPCR validation of key differentially expressed lncRNAs and mRNAs. *p < 0.05.

Five patients with AMI (three patients from RNA sequencing), three patients with MF (two patients from RNA sequencing), and five patients with HF (three patients from RNA sequencing) were included. Based on bioinformatic analysis and literature retrieval results, we selected one differentially expressed lncRNA (PLAC4) and eight differentially expressed mRNA (IL18R1, PFKFB3, IL1R2, IRAK3, FKBP5, LRG1, IKZF2, and RNASE1) for validation (Figure 5). PLAC4 was upregulated in MF vs. AMI, while it was downregulated in HF vs. MF. Besides FKBP5, all above mRNAs were downregulated in MF vs. AMI, while they were upregulated in HF vs. MF. The validated result was basically in line with the RNA sequencing analysis. In addition, the expression validation of IL18R1, IL1R2, LRG1, and RNASE1 in additional HF and MF patients was also performed (Supplementary Figure 1). The method was similar to the description before. The expression trend of these genes was upregulated. Some genes were not expressed significantly. Small sample size may lead to the bias. Larger numbers of samples are further needed.

### Electronic Validation and Diagnostic Analysis of Key Differentially Expressed lncRNAs and mRNAs

**Figure 6:**
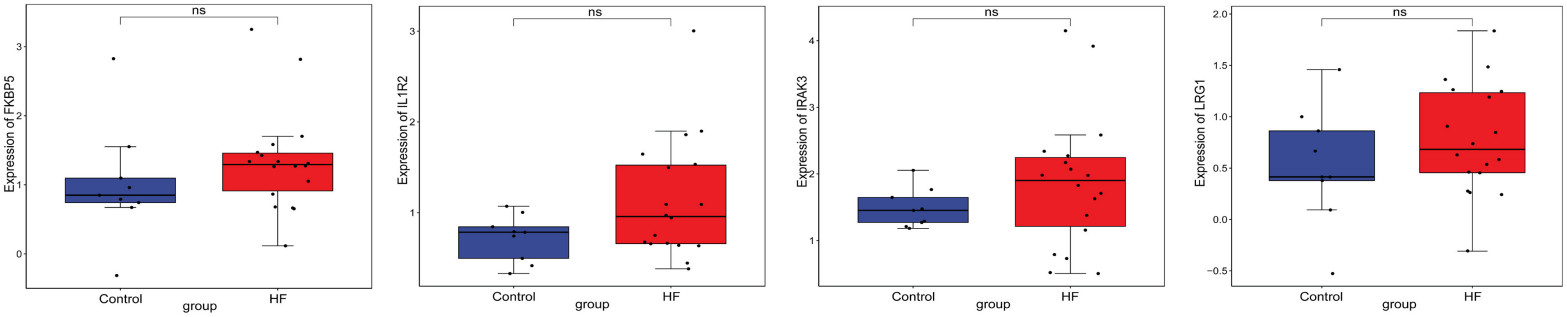
The box plot of FKBP5, IL1R2, IRAK3, and LRG1 in GSE21125 of the HF dataset. Ns, no significant difference.

**Figure 7:**
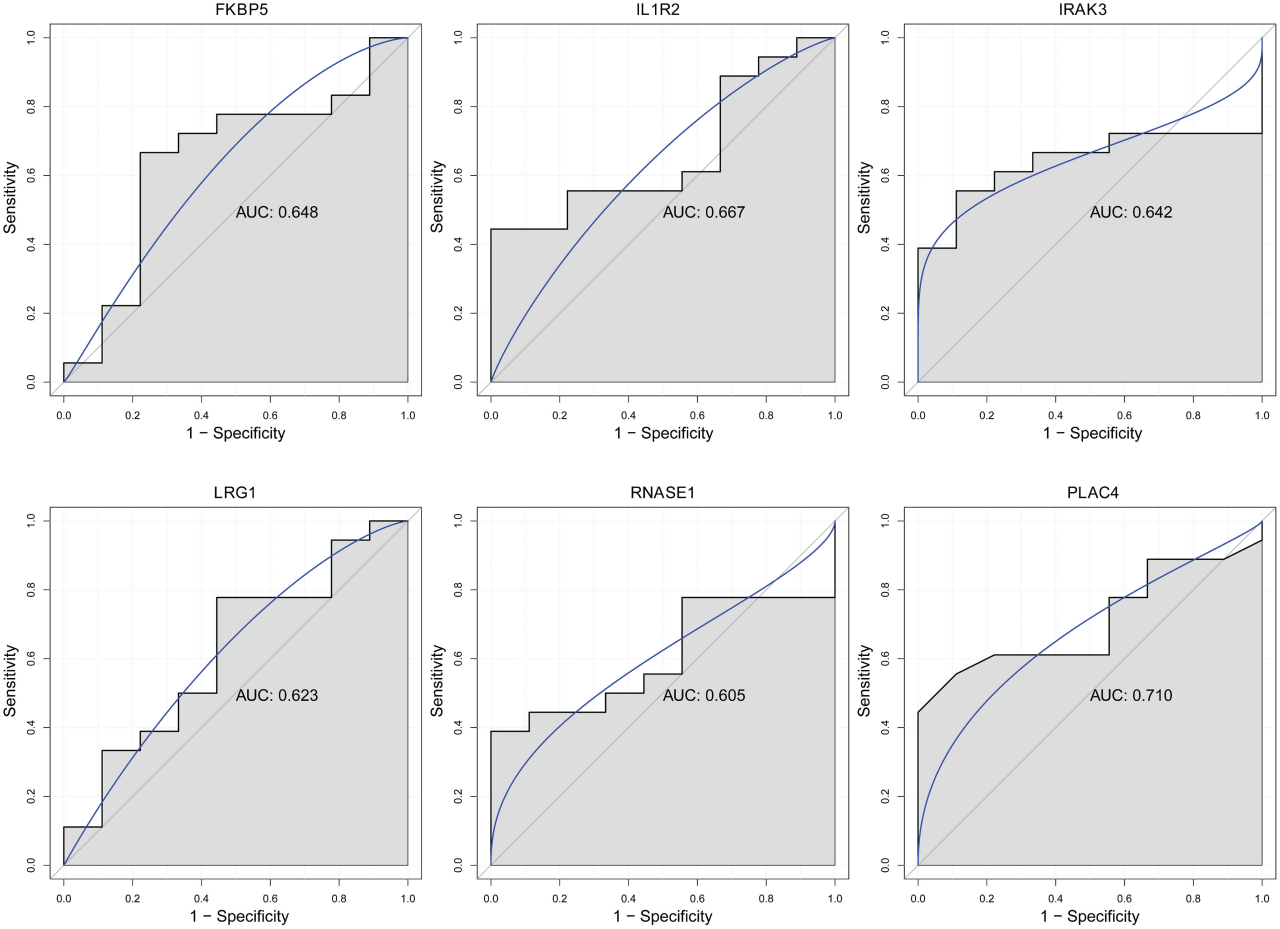
The ROC curves of FKBP5, IL1R2, IRAK3, LRG1, RNASE1, and PLAC4 in HF. The ROC curves were used to show the diagnostic ability with 1 – specificity and sensitivity.

**Figure 8:**
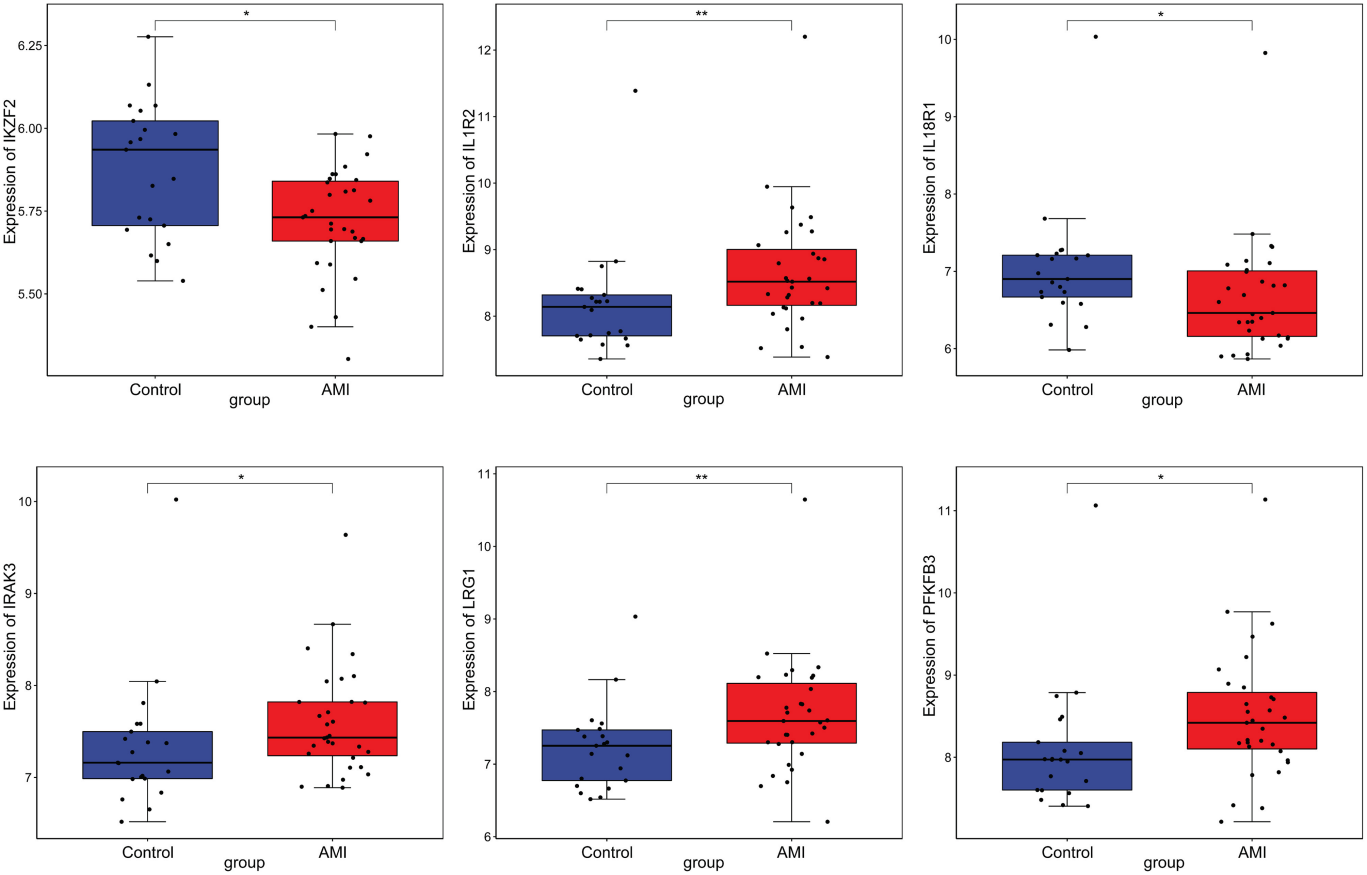
The box plot of IKZF2, IL1R2, IL18R1, IRAK3, LRG1, and PFKFB3 in GSE48060 of the AMI dataset. *p < 0.05; **p < 0.01.

**Figure 9:**
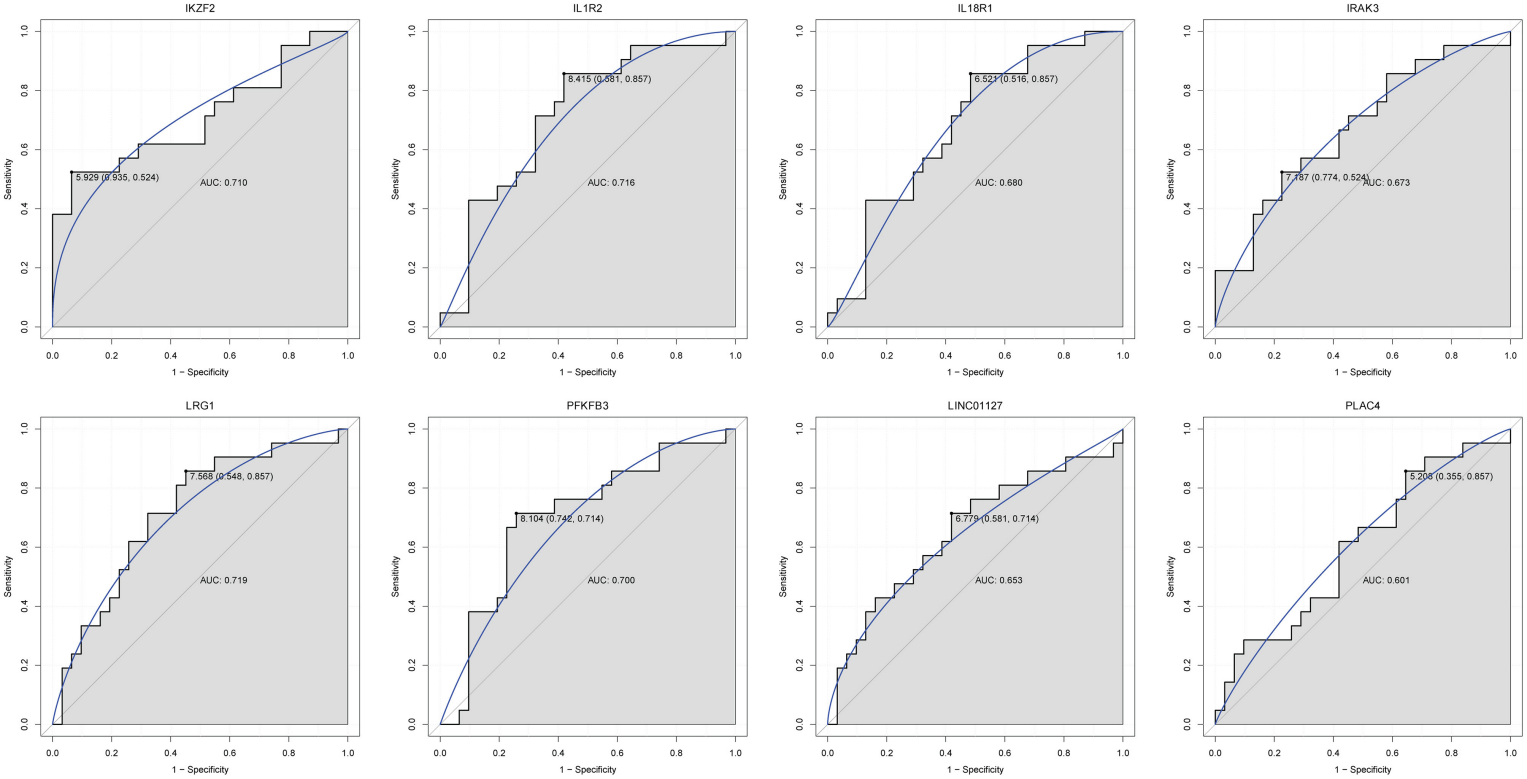
The ROC curves of IKZF2, IL1R2, IL18R1, IRAK3, LRG1, PFKFB3, LINC01127, and PLAC4 in AMI. The ROC curves were used to show the diagnostic ability with 1 – specificity and sensitivity.

Firstly, four key differentially expressed mRNAs including FKBP5, IL1R2, IRAK3, and LRG1 were randomly selected for expression validation in the GSE21125 of HF dataset (Figure 6). FKBP5, IL1R2, IRAK3, and LRG1 were upregulated in HF patients compared with normal controls. This further suggested that upregulation of these mRNAs may play a critical role in the development of HF. In addition, ROC curve analysis was carried out to assess the diagnosis ability of FKBP5, IL1R2, IRAK3, LRG1, RNASE1, and PLAC4 for HF (Figure 7). AUC values of FKBP5, IL1R2, IRAK3, LRG1, RNASE1, and PLAC4 were all more than 0.6, which suggested that they have a potential diagnostic value for HF. Secondly, six key differentially expressed mRNAs including IKZF2, IL1R2, IL18R1, IRAK3, LRG1, and PFKFB3 were randomly selected for expression validation in the GSE48060 of the AMI dataset (Figure 8). IL1R2, IRAK3, LRG1, and PFKFB3 were significantly upregulated in AMI patients compared with normal controls. IKZF2 and IL18R1 were remarkably downregulated in AMI patients compared with normal controls. This indicated that these mRNAs may play a significant role in AMI. In addition, ROC curve analysis was performed to assess the diagnosis ability of IKZF2, IL1R2, IL18R1, IRAK3, LRG1, PFKFB3, LINC01127, and PLAC4 for AMI (Figure 9). AUC values of IKZF2, IL1R2, IL18R1, IRAK3, LRG1, PFKFB3, LINC01127, and PLAC4 were all more than 0.6, which suggested that they have a potential diagnostic value for AMI. It is noted that IL1R2, IRAK3, LRG1, and PLAC4 could be considered as potential diagnostic markers for both AMI and HF.

## Discussion

Up to now, no reports about AC005392.3 have been recorded. The expression level of AC007278.2 is upregulated in peripheral blood mononuclear cells in multiple sclerosis (22). The IL-18 cytokine, coded by interleukin 18 receptor 1 (*IL18R1*), has been found to be involved in atherosclerosis and cardiovascular complications (23). Bochmann et al. found that IL18R1 is a high-confidence marker for epicardial cells (24). The single nucleotide polymorphism of the *IL18R1* gene has been found in cardiovascular disease, such as coronary artery disease (25, 26). In addition, the expression level of IL18R1 is significantly altered in systemic and arterial bed-dependent atherosclerosis plaques (27). IL18R1 is upregulated in patients with acute ST elevation MI (28, 29). In this study, we first found the association between AC005392.3, AC007278.2, and IL18R1. This suggested that AC007278.2 and IL18R1 may be associated with atherosclerosis and cardiovascular complications, which may provide a novel field in understanding the disease progression of AMI, MF, and HF. Moreover, IL18R1 could be considered as a potential diagnostic biomarker for AMI patients.

AL356356.1 is significantly related to the clinical traits of clear cell renal cell carcinoma patients and could serve as a predictor for the disease (30). The highest percentage of candidate fusion transcripts of AL137145.2-6-phosphofructo-2-kinase/fructose-2, 6-biphosphatase 3 (PFKFB3) was found in non-small cell lung cancer (31). In cerebrovascular disease of moyamoya disease, AL137145.2 acts as ceRNAs to regulate C-X-C motif chemokine ligand 16 (CXCL16) through miR-107 (31). It is noted that PFKFB3-regulated glycolysis in endothelial cells also plays an important role in vascular sprouting (32, 33). PFKFB3 also maintains endothelial cells in a quiescent state to reduce injury- and inflammation-induced pathological processes including atherosclerosis (34–36). Higher levels of PFKFB3 have been detected in patients with symptomatic atherosclerosis and incident MI (37, 38). In the present study, we found that PFKFB3 was co-expressed with AL356356.1. Moreover, it is a nearby target mRNA of AL137145.2. Furthermore, PFKFB3 had a potential diagnostic value for AMI patients. Interaction pairs of AL356356.1/AL137145.2-PFKFB3 may be involved in maintaining the function of endothelial cells in the process of AMI, MF, and HF.

Dysregulated MKNK1-AS1 could predict adverse cardiovascular outcomes in patients with end-stage renal disease (39). It is reported that LINC01127 promotes the development of ovarian tumors (40). Upregulation of LINC01127 has been observed in clear cell renal cell carcinoma (41). Interleukin 1 receptor type 2 (IL1R2) is associated with the inflammatory response, which leads to the development of atherosclerotic plaques (42, 43). Additionally, DNA methylation and single nucleotide polymorphism are respectively found in subclinical cardiovascular disease and coronary heart disease (44, 45). It is pointed out that IL1R2 is upregulated in vasculitis and AMI patients (46, 47). Herein, we found the interaction pairs of MKNK1-AS1/LINC01127-IL1R2 in AMI, MF, and HF, which indicated that these lncRNAs and mRNAs play a role in atherosclerosis. In addition, IL1R2 could be considered as a potential diagnostic biomarker for both AMI and HF patients.

Besides interaction with co-expressed/nearby lncRNAs, IL18R1, PFKFB3, and IL1R2 were also significantly involved in some signaling pathways according to the KEGG analysis. For example, IL18R1 was enriched in the TNF signaling pathway and cytokine–cytokine receptor interaction; PFKFB3 was enriched in fructose and mannose metabolism and HIF-1 signaling pathway; IL1R2 was enriched in hematopoietic cell lineage and fluid shear stress and atherosclerosis. The TNF signaling pathway is linked to cardiac remodeling following MI (48). It has been found that TNF-α is involved in the pathogenesis and progression of MF (49). The clinical trial testing of anti-TNF-α drugs has been performed in patients with chronic HF (50, 51). It has been demonstrated that cytokine–cytokine receptor interaction plays an important role in the occurrence and development of AMI and HF (47, 52). Increased activity of fructose and mannose metabolism is found in acute myocardial ischemia injury (53). Parisi et al. found that HIF-1 had the diagnostic potential effect of ischemia, which can effectively reflect the process of AMI to a certain extent (54). Moreover, lack of HIF-1 causes angiogenesis disorders and MF, which leads to HF (55). Hematopoietic stem cells have a potential regenerative ability in the AMI xenotransplantation (56). In addition, hematopoietic cells can trans-differentiate into cardiac fibroblasts when ischemic injury occurs (57). Westenbrink et al. found that HF affected multiple hematopoietic lineages (58). Reduced shear stress and low wall shear stress gradient are considered to increase the possibility of atherosclerosis (59, 60). Atherosclerosis is the most common cause of MI and HF (61, 62). Thus, it can be seen that the abovementioned signaling pathways and enriched genes play an important role in cardiovascular disease.

Between MF vs. AMI and HF vs. MF, we also found several other common differentially expressed lncRNAs (such as PLAC4) and differentially expressed mRNAs including interleukin 1 receptor associated kinase 3 (IRAK3), leucine-rich alpha-2-glycoprotein 1 (LRG1), ribonuclease A family member 1 (RNASE1), FKBP prolyl isomerase 5 (FKBP5), and IKAROS family zinc finger (IKZF2). PLAC4 is associated with congenital heart disease and hypertrophic cardiomyopathy (63, 64). The expression level of IRAK3 is significantly increased 2 days post-MI (65). Upregulation of LRG1 has been found in incident MI (38). In mouse models of MI, *LRG1* ablation leads to aggravate MF and heart dysfunction (66). In addition, LRG1 participates in vascular remodeling during HF (67). RNASE1 is upregulated in patients with a history of early MI (68). It is speculated that RNASE1 could be considered as a novel prognostic biomarker for the process of HF after AMI (69). It has been demonstrated that FKBP5 is upregulated and plays a crucial role in endothelial platelet aggregation in AMI patients (70, 71). IKZF2 is overexpressed in the left ventricular myocardium (72). In this study, we found that IRAK3, LRG1, RNASE1, and FKBP5 were downregulated in MF vs. AMI, while they were upregulated in HF vs. MF. On the contrary, PLAC4 and IKZF2 were upregulated in MF vs. AMI, while they downregulated in HF vs. MF. Thus, it can be seen that these genes may be involved in the function of vascular endothelial cells. In addition, FKBP5 was remarkably enriched in the estrogen signaling pathway. In the serum, estrogen receptor β activation reduces the infarct size and lowers the levels of myocardial enzymes (73). In addition, estrogen attenuates MF in HF mainly *via* estrogen receptor β (74). It is indicated that the estrogen signaling pathway (in which FKBP5 is involved) could reduce the infarct size. It is worth mentioning that PLAC4, IRAK3, and LRG1 had a potential diagnostic value for both AMI and HF in our study.

In summary, identified lncRNA-co-expressed/nearby targeted mRNAs pairs including AC005392.3/AC007278.2-IL18R1, AL356356.1/AL137145.2-PFKFB3, and MKNK1-AS1/LINC01127-IL1R2 and several signaling pathways (TNF signaling pathway, cytokine–cytokine receptor interaction, fructose and mannose metabolism, HIF-1 signaling pathway, hematopoietic cell lineage, fluid shear stress and atherosclerosis, and estrogen signaling pathway) may be involved in the progression from AMI to MF to HF. Significantly, IL1R2, IRAK3, LRG1, and PLAC4 had a potential diagnostic value for both AMI and HF. Our study may provide a theoretical basis for the diagnosis and mechanism of research for the development process of AMI–MF–HF patients, which can be valuable for patient prognosis.

However, there are limitations to our study. Firstly, the sample size in RNA sequencing and qPCR is small. Large numbers of blood samples are further needed for RNA sequencing and *in vitro* validation. Secondly, we did not validate the expression of identified lncRNAs and mRNAs and explore deeper pathologic mechanisms of the disease progression. More functional and biological validation experiments, such as immunostaining, ELISA, or Western blot, are further needed. In addition, further mice model experiments are needed to validate and investigate the detailed molecular mechanism of these lncRNAs and mRNAs.

## Data Availability Statement

We have deposited the RNA seq data in a public, community-supported repository of GEO (GSE168281).

## Ethics Statement

The studies involving human participants were reviewed and approved by Shijiazhuang People's Hospital. The patients/participants provided their written informed consent to participate in this study.

## Author Contributions

EW, QC, and YY analyzed the data. LX, XZ, RW, and ZW interpreted the data. SW was a major contributor in writing the manuscript. XH designed the project. All authors read and approved the final manuscript.

## Conflict of Interest

The authors declare that the research was conducted in the absence of any commercial or financial relationships that could be construed as a potential conflict of interest.

## Supplementary Material

Supplementary Figure 1 The *in vitro* qPCR validation of IL18R1, IL1R2, LRG1, and RNASE1 in additional HF and MF patients. ^*^*p* < 0.05.
