# Supplementary figures and images for: Uncovering Potential lncRNAs and mRNAs in the Progression From Acute Myocardial Infarction to Myocardial Fibrosis to Heart Failure
Source: Front Cardiovasc Med. 2021 Jul 16;8:664044. doi: 10.3389/fcvm.2021.664044 (PMC8322527; doi:10.3389/fcvm.2021.664044)

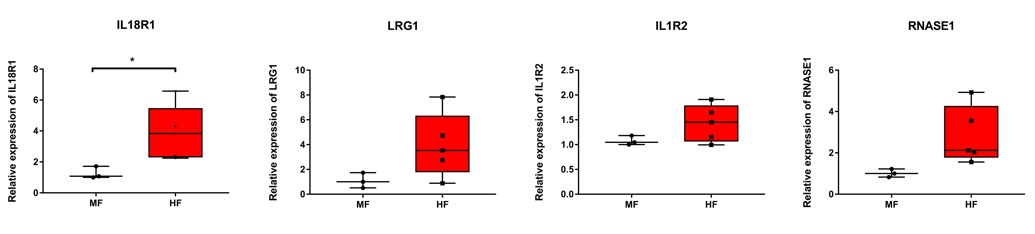

Supplement: Supplementary Figure 1 — The in vitro qPCR validation of IL18R1, IL1R2, LRG1, and RNASE1 in additional HF and MF patients. *p < 0.05. [file Image_1.PNG]
